# SMILE: a novel procedure for subcellular module identification with localisation expansion

**DOI:** 10.1049/iet-syb.2017.0085

**Published:** 2018-04-01

**Authors:** Lixin Cheng, Pengfei Liu, Kwong‐Sak Leung

**Affiliations:** ^1^ Department of Computer Science & Engineering Chinese University of Hong Kong Ma Liu Shui Hong Kong

**Keywords:** cellular biophysics, proteins, molecular biophysics, subcellular module identification, localisation expansion, computational clustering methods, protein‐protein interaction network, biological functions, protein interaction structure, protein subcellular localisation, subcellular modules, InWeb‐InBioMap datasets, subcellular localisation

## Abstract

Computational clustering methods help identify functional modules in protein–protein interaction (PPI) network, in which proteins participate in the same biological pathways or specific functions. Subcellular localisation is crucial for proteins to implement biological functions and each compartment accommodates specific portions of the protein interaction structure. However, the importance of protein subcellular localisation is often neglected in the studies of module identification. In this study, the authors propose a novel procedure, subcellular module identification with localisation expansion (SMILE), to identify super modules that consist of several subcellular modules performing specific biological functions among cell compartments. These super modules identified by SMILE are more functionally diverse and have been verified to be more associated with known protein complexes and biological pathways compared with the modules identified from the global PPI networks in both the compartmentalised PPI and InWeb_InBioMap datasets. The authors’ results reveal that subcellular localisation is a principal feature of functional modules and offers important guidance in detecting biologically meaningful results.

## 1 Introduction

Protein–protein interaction (PPI) resources are invaluable for proteomic and network related analysis, which have provided great insights for the mechanistic understanding of human diseases and drug design [1–9]. Computational clustering methods help identify functional modules in PPI networks since proteins usually cluster together to participate in the same biological pathways or specific functions [10–14]. However, cell compartmentalisation is overlooked by previous standard procedures, despite a large number of exciting results emerging from analyses at the global cellular level [15–18]. In fact, eukaryotic cells are composed of several subcellular compartments that enable the cell to implement various metabolic activities simultaneously, and proteins need to target appropriate compartment to interact with each other and to form compound functional complexes in the signalling pathways for specialised biological processes and functions [19, 20].

Cell compartmentalisation, the formation of cellular compartments, is physical and a vital regulator of several main biochemical processes in eukaryotic cells, which assign certain biomolecules in different partitions of the cell. Several properties and characteristics like intracellular pH and enzyme systems distinguish one compartment from the others [21]. Many works have observed that the interactions and functions of proteins are closely related to their localisation in the cell [22]. More importantly, localising in common compartments is of vital importance for proteins to interact with each other, at least transiently or conditionally. Accumulated experimental evidence suggested that translocation is an efficient regulation mode in cells and erroneous localisation may lead to disorders or even diseases [23, 24]. For instance, a transcription factor *P53* may be located to a nucleus to promote transcription of certain genes and thereby activating autophagy program upon stimulation, a cellular process of self‐eating [25–28]. In contrast, when targeting at cytoplasm, however, *P53* plays an opposite role as a master repressor of the autophagy program [29, 30]. Moreover, localisation‐based modulation can change cellular program completely. For instance, the protein *ATG5* is involved in several cellular processes including autophagy and apoptosis. The two cellular programs can be switched as the localisation of *ATG5* changes between mitochondria and cytoplasm [31]. These common examples in experimental biology cannot be fully figured out through analysing the global cellular network without a comprehensive study of analysing the localisation compartmentalised subnetworks. Therefore, identifying modules consisting of closely interacted proteins localised in a specific compartment is expected to generate more biologically meaningful results, as cells can naturally be decomposed into several compartments.

In the meanwhile, several algorithms have been designed for the identification of protein complexes in the bioinformatics community, although none of them take the cell compartmentalisation into consideration. ClusterONE (Cluster with Overlapping Neighbourhood Expansion) [16] strives to discover not only densely connected clusters with comparable accuracies but also possibly overlapping clusters. It executes a greedy growth algorithm to cluster networks from small seeds supervised by a fitness function concentrating on the cluster separability, which is formulated by the ratio between the number of internal interactions of a cluster and the number of all interactions linking the cluster. Then each generated cluster is statistically evaluated by a probability using Monte Carlo random interaction number of the clusters. Another clustering algorithm focusing on the explicit topological structure of protein complexes is finding low‐conductance sets with dense interactions (FLCD) [32], a two‐step algorithm considering both the internal and external connectivity of protein complexes. It first detects clusters with high separability and then the clusters with high edge density are detected as protein complexes. By mimicking Markovian random walk on networks, several other clustering algorithms were also developed, such as Markov Clustering (MCL), regularized Markov Clustering (R‐MCL), and soft regularised Markov Clustering (SR‐MCL) [12, 33]. MCL simulates many stochastic flows within a network by making the strong flows stronger and the weak ones weaker. After multiple iterations, the identified cluster come out with strong internal flows and separated by the boundary with no flows [33]. R‐MCL, an improved version of MCL which is more accurate and less time consuming, scales much better to moderate sized networks by penalising the large clusters at each iteration. However, both R‐MCL and SR‐MCL can only identify non‐overlapped clusters. To address this problem, another method SR‐MCL was developed to achieve overlapped clusters by executing R‐MCL multiple times [12].

Additionally, it is worth pointing out that proteins are interacting spatially to form a dynamic cellular network. Some proteins are localised in multiple compartments and may not directly interact with each other in the same compartment, but they still work towards similar cellular functionalities and hence should belong to the same modules. For instance, transmembrane receptor proteins tend to interact with cytoplasmic proteins as well as with extracellular ligands in signal transduction cascades [34]. Hence, highly overlapped protein modules, either from the same or different compartments, need to be merged to achieve the final super modules.

In this study, we introduce a novel procedure, subcellular module identification with localisation expansion (SMILE), to identify subcellular modules from each cell compartment with localisation extension. Theoretically, the identified super modules engage interactions with high confidence. Experimentally, our results demonstrate that SMILE outperforms the conventional clustering method with respect to protein complex detection and biological pathway annotation, especially for the novel modules exclusively identified by SMILE.

## 2 Material and methods

### 2.1 Datasets

Two human PPI networks, ComPPI v1.1 [24] and InWeb_ InBioMap 2016_09_12 [15], were employed to identify the functional modules in this study. ComPPI (compartmentalised PPI) is an online database which provides qualitative information on both the interactions among proteins and their localisations. With experimental evidence, the interactions in ComPPI are collected from nine high‐quality PPI databases, i.e. the Drosophila Interactions Database (DroID), the Human Protein Reference Database (HPRD), the Matrix Database (MatrixDB), the Munich Information Center for Protein Sequences (MIPS), the Biological General Repository for Interaction Datasets (BioGRID), the Center for Cancer Systems Biology (CCSB), the Database of Interacting Proteins (DiP), the IntAct Molecular Interaction Database (IntAct), the Molecular INTeraction Database (MINT). We excluded the biological unlikely interactions with interaction scores <0.8, resulting in 16,053 proteins and a total of 193,691 corresponding interactions among six major cellular compartments, i.e. nucleus, cytosol, mitochondrion, secretory‐pathway, membrane, and the extracellular compartment. The major compartments were defined in the ComPPI database, among which several minor secretory organelles are combined into one major compartment ‘secretory‐pathway’, including Golgi apparatus, endoplasmic reticulum, endosome, peroxisome, lysosome, vacuole, and vesicles. The subcellular localisation annotations are coming from both experiments and computational predictions. The same as Veres *et al.*, localisation score is used to measure the probability of localisation for each protein, depending on the evidence type (experimental or predicted) and the number of sources. Only proteins with a high localisation score (>0.8) are retained for further study.

InWeb_InBioMap, or simply InWeb_IM for short, is the largest dataset of human PPIs at present. It has an extremely large coverage of PPIs (more than half a million) that are retrieved from eight orthology PPI datasets. 57% of the interactions have experimental evidence and the others were computational predicted. Similar to Veres *et al.* [24] suggested we assigned the localisation information to proteins and interactions by calculating the localisation score and interaction score, respectively. The interaction score distribution of ComPPI shows a majority of interactions score higher than 0.8 as shown in Figure S1, so we can end up with the same results with other thresholds less than it.

To evaluate the performance of SMILE on protein complex detection, we estimated the identified modules with two golden standards of a protein complex, i.e. a comprehensive resource of mammalian protein complexes and Protein Complex Database with a Complex Quality Index (PCDq) [35–37]. The latest versions of them were used and only protein complexes including five or more members were considered for further study.

To examine whether the identified modules are biologically meaningful, we used four pathway resources, Kyoto Encyclopedia of Genes and Genomes (KEGG) [38, 39], Protein ANalysis THrough Evolutionary Relationships (PANTHER) [40, 41], BioCarta (https://cgap.nci.nih.gov/Pathways /BioCarta_Pathways), and Reactome [42, 43], as the golden standards for function prediction. Pathway annotations of PANTHER were obtained from PANTHER Pathway 3.4.1 and the other data were collected from the curated gene sets of Molecular Signatures Database (MSigDB v5.2) [44, 45].

### 2.2 Global module identification

We used ClusterONE [16] to identify modules from the entire PPI network. ClusterONE strives to discover not only densely connected clusters with comparable accuracies but also possibly overlapping regions within a given network, a distinct advantage of ClusterONE. It plugs in Cytoscape [46] and executes a greedy growth algorithm to cluster networks from small seeds supervised by a fitness function. Each generated cluster is evaluated by a cohesiveness score, which is a ratio of the practical interaction number over the theoretical interaction number of the cluster, measuring how likely is a group of proteins to be a module (or cluster separability) [16]. Let *V* denote a cluster in the PPI network, $w^{\text{in}}(V)$ denotes the number of interactions contained within the cluster, $w^{\text{bound}}(V)$ denotes the number of interactions coming out of the cluster, and p|V| is a penalty term aiming to model the uncertainty of unchecked interactions in the PPI network, the cohesiveness of *V* is defined as follows:

(1) $$f\left(V\right)=\frac{w^{\text{in}}(V)}{w^{\text{in}}\left(V\right)+w^{\text{bound}}\left(V\right)+p|V|}$$

In this study, we used the default function parameters of ClusterONE and only the identified clusters with a size larger than ten were considered as modules, as small modules are usually more factorisable [47]. These clusters were defined as global modules since they were identified in the entire PPI network instead of the compartmentalised subnetworks.

### 2.3 Super module identification

**Fig. 1:**
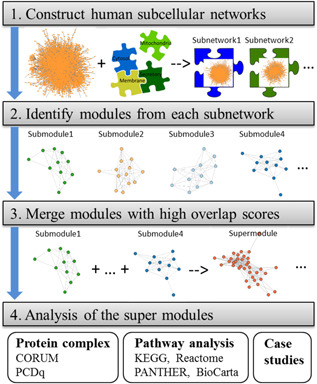
Flowchart of the main steps for super module identification

As shown in Fig. 1, the procedure of SMILE works in three steps: first, constructing subcellular networks based on localisation annotation; second, identifying clusters with high cohesiveness from each subnetwork; and third, combining highly overlapped clusters.

(i) *Subnetwork construction:* Suppose the input PPI network is G=(P,E), where *P* is the set of proteins and *E* is the set of interactions among proteins. Each protein *P* is annotated to one or more than one compartment. Based on the information of protein subcellular localisation, we extract subnetworks where proteins are localised in an identical compartment. For compartment $C_{i}$, we can define its corresponding subnetwork to be $G_{i}=(P_{i},E_{i})$, where $P_{i}$ is the subset of proteins in the compartment $C_{i}$ and $E_{i}$ is the subset of interactions in the compartment $C_{i}$.
(ii) *Module identification in each subnetwork:* Calculate the cohesiveness of $f(V_{i})$ (see (1)) in compartment $C_{i}$ using ClusterONE, where $V_{i}$ is the clusters in the subnetwork of $C_{i}$.
(iii) *Merging:* The highly overlapped modules identified from different subcellular networks are merged together to generate super modules. Let *A* and *B* represent two clusters of proteins from different subnetworks, respectively. Their similarity is measured by an overlap score, which is defined as
(2) $$\omega \left(A,B\right)=\frac{|A\cap B{|}^{2}}{\left|A||B\right|}$$
  where |A| and |B| are the sizes of the two clusters, respectively, and |A∩B| is the number of the overlapping proteins annotated with both subcellular locations.

**Fig. 2:**
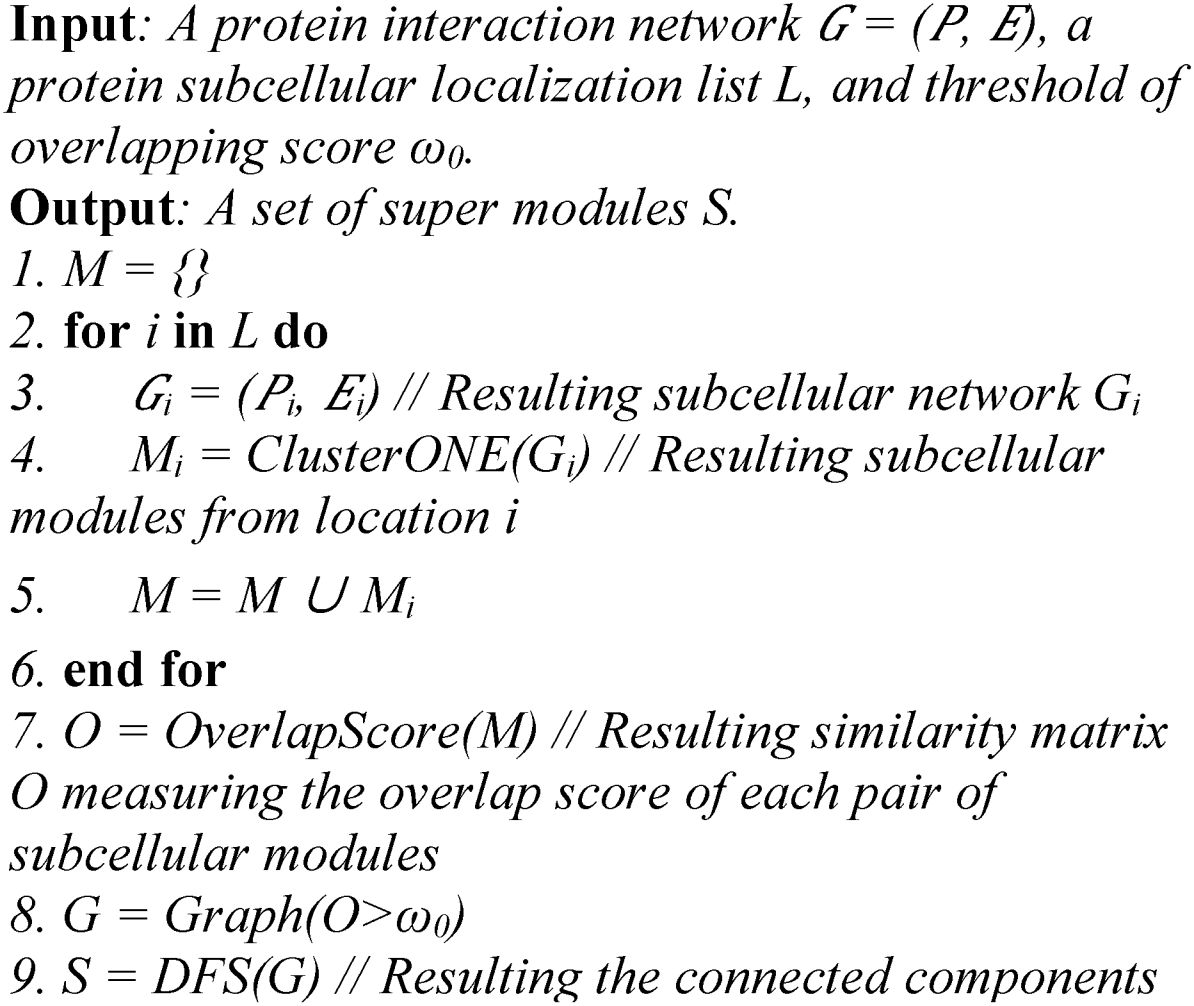
Algorithm: SMILE procedure

Lastly, we combine subcellular modules identified from different subnetworks. Other than the module compartment, the proteins may localise in some other compartments, considering the multi‐localisation property of proteins. We first calculate the overlap scores for each pair of subcellular modules and constructs an adjacency matrix in which each row (or column) represents a module. Two modules are overlapped if the overlap score is larger than a given threshold $\omega_{0}$. The threshold is set as 0.5 by default, which implies >70% of the members of the two modules being compared are overlapped if they have the same size. Then, we create a graph from the adjacency matrix and split it into connected subgraphs (or components) using depth‐first search, where nodes correspond to modules and edges denote the overlapping relationship among all modules. Finally, the modules in each subgraph are merged together and defined as a super module. Mathematically, the procedure of SMILE is also shown in the box of algorithm (see Fig. 2). The identified super modules are essentially all connected components of subcellular modules.

### 2.4 Section headings

To evaluate the performance of SMILE, we used three quality measurements [11, 13, 16] to compare the results of golden standard complexes with the global modules (modules found using ClusterONE), the super modules (modules found by our method SMILE), and novel modules (modules in a super module but do not in global module). Given *r* predicted and *s* reference complexes, let $t_{ij}$ denote the number of proteins that exist in both predicted complex *i* and reference complex *j*, $v_{i}$ and $w_{j}$ represent the number of proteins in predicted complex *i* and reference complex *j*, respectively. Then the three measurements, Sn (sensitivity), PPV (positive predictive value), and Acc (accuracy), are defined as follows:

(3) $$\text{Sn}=\frac{\sum_{j=1}^{s}\max_{i=1,\ldots r}t_{ij}}{\sum_{j=1}^{s}w_{j}}$$

(4) $$\text{PPV}=\frac{\sum_{i=1}^{r}\max_{j=1,\ldots s}t_{ij}}{\sum_{i=1}^{r}\sum_{j}^{s}t_{ij}}$$

(5) $$\text{Acc}=\frac{\sum_{j=1}^{s}\max_{i=1,\ldots r}t_{ij}}{\sum_{i=1}^{r}v_{i}}$$

Essentially, Acc is the geometric mean of Sn and PPV. Using the three measurements, we evaluated the global modules, super modules and novel modules with two reference sets CORUM and PCDq (see Section 2.1), respectively.

### 2.5 Evaluation of module biological relevance

The hypergeometric test was adopted to evaluate whether a module, *M*, is overrepresented within a biological pathway, *X*. The probability of observing at least *t* proteins annotated by *X* with size *T* is defined as

(6) $$P=\overset{n}{\underset{i=t}{\sum }}\frac{\left(\begin{array}{c} N-T\\ n-i \end{array}\right)\left(\begin{array}{c} T\\ i \end{array}\right)}{\left(\begin{array}{c} N\\ n \end{array}\right)}$$

where *N* is the total number of proteins in the given PPI network and *n* is the size of the module *M*. The outputting *P* value is then adjusted by the Benjamini & Hochberg method for false discovery rate control. The pathway is said to be enriched in the module *M* at a significance level if the adjusted P<0.05.

The overrepresentation score (ORS) [13, 16] was used to evaluate the biological relevance of the identified modules in pathways. We say a module is biologically meaningful if it is significantly enriched in any biological pathway. Given a set of identified modules, ORS is calculated as the ratio of the number of biologically meaningful modules over the size of the module set, given as

(7) $$\text{ORS}=\frac{\sum_{i}^{U}\mathrm{sgn}\left(\sum_{j}^{V}\mathrm{sgn}\left(P_{\text{cutoff}}-P_{M_{i}X_{j}}\right)-1\right)}{U}$$

where *U* is the total number of identified modules and *V* equals the number of pathways. $P_{M_{i}X_{j}}$ represents the adjusted *P* value for module *M* and pathway *X*, while $P_{\text{cutoff}}$ represents the threshold of the *P* value of hypergeometric test. ORS ranges from 0 to 1, where 1 represents the case that all the identified modules are significantly associated with reference pathways.

## 3 Results

### 3.1 Super modules and novel modules

**Fig. 3:**
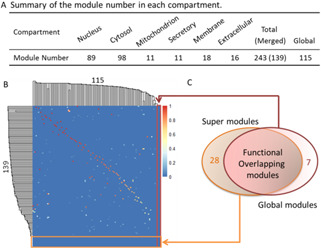
Overview of the module numbers and the comparison between super modules and global modules in ComPPI ***(a)*** Number of identified subcellular modules (243), super modules (139), and global modules (115), ***(b)*** Heatmap of the overlap scores between super modules and global modules. It is essentially an adjacent matrix between the two module sets, in which red represents high score while blue represents low score, ***(c)*** Venn diagram for the super modules and global modules. The super modules involve 28 novel modules and the global modules contain seven unique modules

We used ClusterONE to predict functional modules from the ComPPI network. As shown in Fig. 3, a total of 115 modules are identified and defined as global modules. Using SMILE, on the other hand, we identified 89, 98, 11, 11, 18, and 16 individual modules from nucleus, cytosol, mitochondrion, secretory‐pathway, membrane, and extracellular, respectively. These subcellular modules were then merged to generate super modules with larger size if they share substantial module members (see Methods and Figure S2). Eventually, we obtained 139 super modules and 28 out of them have no functional overlap with the global ones (overlap score <0.25). For simplicity, hereafter the super modules not functionally overlapped with the global modules were called as *novel modules*. Likewise, the modules involved in the global module set but not included in the super module set are defined as *global unique modules*. For the InWeb_IM network, as shown in Figure S3, 261 super modules and 158 global modules were captured using SMILE and ClusterONE, respectively. Among the super modules, around one‐third of them (82) are novel modules. Note that here the overlap score threshold $\omega_{0}$ is set as 0.5 by default since we have no preference to merge the modules with high or low overlap for the human PPI datasets. However, for species with quite complete PPI networks, such as yeast, a threshold of 0.75 is suggested to guarantee only highly overlapped modules are merged. Additionally, a series of the overlap score thresholds were used to study the parameter sensitivity of $\omega_{0}$. Please refer to Supplementary Table S2 for more details.

### 3.2 Performance comparison for protein complex identification

**Fig. 4:**
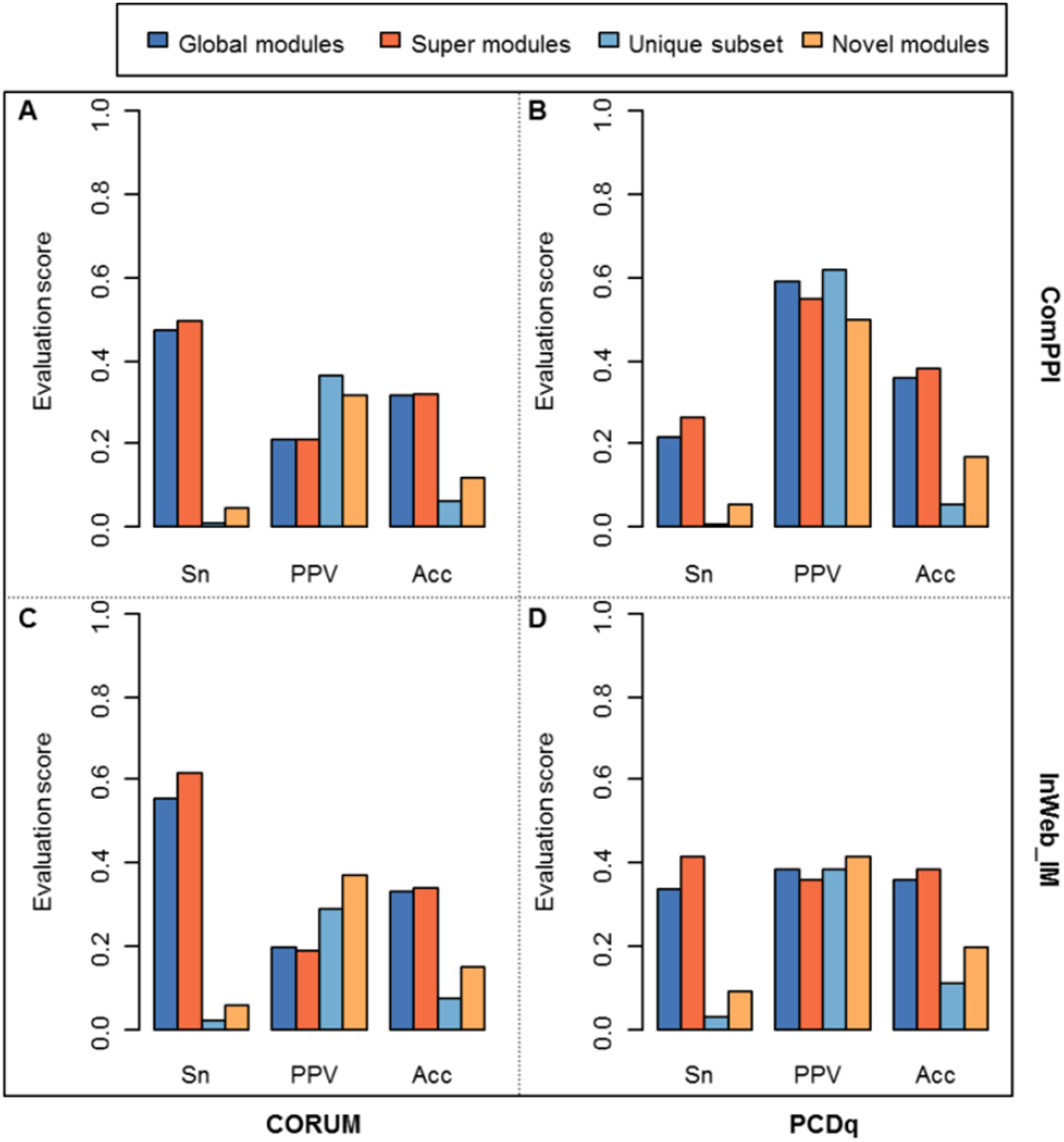
Performance of the protein complex prediction. Two PPI networks and two complex references were used for evaluation ***(a)*** Results using ComPPI network and CORUM reference, ***(b)*** Results using ComPPI network and PCDq reference, ***(c)*** Results using InWeb_IM network and CORUM reference, ***(d)*** Results using InWeb_IM network and PCDq reference. Sn, sensitivity; PPV, positive predictive value; Acc, accuracy

We found SMILE outperforms the conventional procedure of ClusterONE based on two protein complex reference sets, CORUM and PCDq, on two PPI networks ComPPI and InWeb_IM. Three measurements, Sn, PPV, and Acc, were used to assess the quality of the identified module sets with respect to protein complex prediction. As shown in Fig. 4, it is clear that the super modules generated by SMILE have the highest Acc score and cover more proteins clustered into the reference complexes or modules on both networks. For the ComPPI network, SMILE consistently gets higher Sn and Acc scores based on both references and can identify a comparable proportion of matched complexes in CORUM. Although ClusterONE has a higher PPV than SMILE when using PCDq as the golden standard, SMILE is able to detect more matched protein complexes (76 versus 68, Table S1). For the InWeb_IM network, we can achieve the similar result for the performance of SMILE, which consistently achieves the highest scores of Sn and Acc and a comparable PPV. Strikingly, the exclusively identified novel modules tend to match more protein complexes from both CORUM and PCDq. In particular, 0.3701 and 0.4139 of the novel modules are highly associated with reference complexes from CORUM and PCDq, respectively, whereas the figures are only 0.2885 and 0.3820 for the ClusterONE unique modules. It makes an opposite result in the ComPPI dataset for PPV instead, the reason might be that the coverage of ComPPI is much lower than InWeb_IM and therefore a larger number of unpredicted interactions have yet to be addressed. For more details please refer to Table S1.

The ORSs (see (7)) are calculated for the three types of modules of the two networks, respectively, among four pathway resources, KEGG, PANTHER, BioCarta, and Reactome. The composite score is the sum of ORS for the four resources.

### 3.3 Performance comparison for biological pathway annotation

**Fig. 5:**
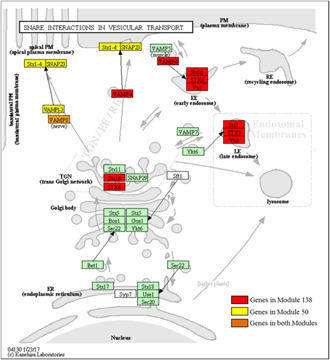
SNARE interactions in vesicular transport pathway in KEGG. Genes clustered in modules 50, 138, and both, are marked in yellow, red and orange, respectively. Module 50 is a super module while module 138 is a novel module

Then, we examined the biological relevance of the detected modules by performing overrepresentation analysis of pathway associations. As shown in Table 1, super modules, especially the novel ones, consist of more proteins in the biological pathways on all the four resources on both PPI networks of ComPPI and InWeb_IM. Specifically, in ComPPI, 106 out of 139 super modules (76.26%) are significantly over represented in the KEGG pathways, while this number is dropped to 83 (72.17%) for the modules identified using ClusterONE. For the novel identified modules, 75% of them are significantly enriched in the KEGG pathways, which is also higher than the figure of global modules and its unique subset. In particular, Fig. 5 illustrates the KEGG pathway, ‘SNARE interactions in vesicular transport’ (hsa04130), comprise a significant proportion of proteins that are predicted as members of super modules. Specifically, the proteins in two super modules, module 26 and module 51, were mapped to the KEGG pathways [38, 39] using the KEGG Mapper facility (http://www.genome.jp/kegg/mapper.html). The proteins involved in modules 26 and 51 are marked in yellow and red, respectively, while proteins contained in both modules are marked in orange. It is clear that all the members of module 26 and six proteins in module 51 are the important components of the KEGG PATHWAY: hsa04130, despite the size of the two modules are merely 10 and 15. Strikingly, module 26 is a novel module that is exclusively identified using SMILE, implying its powerful ability in mining pathway relevant information.

**Table 1:** Performance comparison for pathway annotation from four resources

Module set	KEGG	PANTHER	BioCarta	Reactome	Comp score
ComPPI					
global	0.7217	0.2081	0.313	0.9391	2.1819
unique	0.5714	0.2857	0.1428	0.7143	1.7142
super	**0.7626**	0.3165	0.3165	**0.9496**	2.3452
novel	0.75	**0.3929**	**0.3929**	0.9286	**2.4644**
InWeb_IM					
global	0.7975	0.4177	0.3228	0.9241	2.4621
unique	0.6667	0.3333	0.2667	0.8	2.0667
super	0.8314	0.4444	0.3716	0.9579	2.6053
novel	**0.8537**	**0.4878**	**0.3902**	**0.9634**	**2.6951**

The tendency is even more apparent for the InWeb_IM network and other pathway resources. SMILE identified modules, especially the novel modules among them, consistently obtain the highest ORS (Table 1). The composite score is the sum of all ORSs among the four pathway resources and it was used to compare the overall performance of pathway annotation. As expected, the novel modules and super modules consistently obtained the highest and the second highest composite score, both of which are higher than that of the global modules and its unique subset.

### 3.4 Super modules tend to have more biological relevance

**Fig. 6:**
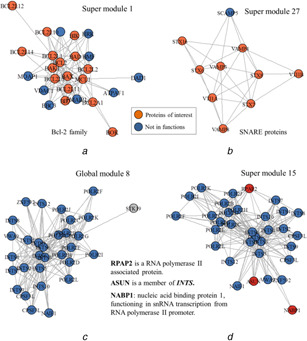
Super modules identified using SMILE are more biologically relevant ***(a)*** Novel module involved in Bcl‐2 family, the proteins in the module are localised in nucleus and cytosol, ***(b)*** Novel module enriched SNARE protein and all its proteins are localised in the extracellular region. Proteins of interest are marked in orange, ***(c‐d)*** Comparison of a global module and its corresponding super module. Blue nodes denote the common proteins involved in both modules, red nodes denote proteins exclusively detected in the super module, and grey nodes denote the global module unique proteins

The novel modules identified by SMILE are not only likely to over‐represent in specific biological pathways, but also tend to involve in gene families. As shown in Fig. 6*a*, the novel module exclusively identified by SMILE in ComPPI consists of 23 proteins and 15 out of them belong to the Bcl‐2 family, an apoptosis regulator filled with evolutionarily related proteins. The proteins in this family supervise mitochondrial outer membrane permeabilisation and usually work as promoters or suppressors of apoptosis [48, 49]. Fig. 6*b* shows another novel module that is enriched with SNARE proteins. Nine out of ten proteins in the module are involved in the protein family of SNARE. The SNARE proteins play a key role as the mediator of vesicle fusion, the fusion of vesicles and their target compartments, among an assortment of others. These results reveal that the SMILE‐identified novel modules are highly associated with biological functions, especially the compartment related functions.

Also, SMILE outperforms ClusterONE with respect to the functionally overlapping modules, the super modules that share a large fraction of proteins with global modules. Fig. 6*c* and *d* illustrates a super module and its corresponding highly overlapped global module captured from the ComPPI network. The super module covers all the members of the global module except STK19, a protein with unknown specific function. These common proteins are mainly involved in two protein complexes, POLR2 (RNA polymerase II) and INTS (Integrator complex), which are in charge of regulating RNA polymerase II and RNA processing. POLR2 is an enzyme that can promote the transcription of DNA to synthesise the precursors of mRNA, snRNA, and microRNA [50, 51]. INTS is a highly conserved nuclear complex that usually interacts with the C‐terminal tail of the largest subunit of the POLR2 complex to promote 3′‐snRNA processing [52]. Interestingly, three more proteins exclusively detected in the super module, ASUN, RPAP2, and NABP1, are closely related to RNA polymerase II. More specifically, ASUN is a member of the complex INTS, RPAP2 is an RNA polymerase II associated protein, and NABP1 functions in snRNA transcription from RNA polymerase II promoter [52, 53]. Overall, SMILE is superior in identifying super modules, either overlapped with global modules or not, with higher biological relevance and functional significance.

### 3.5 Application to MCL

By default, SMILE use ClusterONE to detect functional modules in a given biological network, because ClusterONE is an efficient algorithm that allows identification of overlapping modules and its plugin in Cytoscape is user‐friendly. However, SMILE can be easily applied to other clustering algorithms such as MCL [33], which identifies modules in networks using a mathematical bootstrapping procedure. Hence, MCL was adopted for module identification on both PPI datasets, although it cannot handle overlapping modules. In ComPPI, 28 and 234 modules were identified using MCL and SMILE‐MCL (MCL under the strategy of SMILE), respectively; among them, 200 modules were exclusively selected using SMILE‐MCL while the counterpart is only 2 for MCL. Importantly, based on CORUM and PCDq references, results of MCL revealed that complexes detected with the SMILE strategy consistently have higher scores on all the evaluation scores including Sn, PPV, and ACC. The two algorithms are comparable when comparing pathway ORSs. For the ComPPI dataset, the ORS of SMILE‐MCL for KEGG is less than that of MCL, but SMILE‐MCL outperforms MCL on the other three pathway references. For the PPI dataset of InWeb_IM, almost all the ORSs of SMILE‐MCL are to some extent less than the scores of MCL. The reason is that only 38 modules are identified using MCL, while the number is 246 for the SMILE‐MCL modules, indicating that it is not a powerful way to detect modules merely using MCL.

## 4 Conclusions

Considering the importance of cell compartmentalisation, we propose a novel procedure SMILE for identifying functional protein modules, which first predict modules separately from each cell compartment, and then compound the highly overlapped ones to generate super modules. These super modules derived by SMILE demonstrated better correspondence with known protein complexes on two databases and biological pathways in four resources than the results of conventional procedures.

Although the dataset used in this study has integrated several available data sources to improve data coverage and quality, the method is limited to those proteins with subcellular localisation information. This limitation can be partially addressed using prediction tools, but in the future, much more work is needed to improve the accuracy of these tools.

Taking the protein subcellular location information into account is the major part of the SMILE procedure and it is a general transformation that universally helps existing complex prediction algorithms perform better, although only ClusterONE and MCL were compared in this study. Specifically, ClusterONE outperforms MCL based on the reported performance evaluation scores in the main manuscript and the Supplementary tables. As shown in Table S3, the super modules identified using ClusterONE consistently obtain the highest evaluation scores except for the pathway composite score of the ComPPI data. For the ComPPI dataset, the accuracy scores of ClusterONE are 0.3212 and 0.3978 for the two references CORUM and PCDq, respectively, both of which are much higher than the other accuracy scores calculated from MCL. The pathway composite score of ClusterONE is 2.3452, which is also comparable to that of MCL (2.3547). Better yet, for the InWeb_IM dataset, the ClusterONE induced super modules to show the best performance amongst the others regardless of the clustering strategies and algorithms.

Not limited to ClusterONE and MCL, SMILE is also applicable to other module identification algorithms depending on users’ preference, since it provides more meaningful biological data by evaluating how within a compartment or cross‐compartment protein interactions altered or propagated within proteomic datasets. Furthermore, SMILE can be easily applied to other types of network studies to capture modules with multiple components like lncRNA, miRNA, and mRNA [54–57]. In future studies, we will provide more computational procedures to both the coding and non‐coding molecules to build a more comprehensive picture of how compartmentalised networks can interact.
